# Hepato- and nephroprotective effects of bradykinin potentiating factor from scorpion (Buthus occitanus) venom on mercuric chloride-treated rats

**DOI:** 10.17179/excli2016-777

**Published:** 2016-12-14

**Authors:** Muhammad M. A. Salman, Ahmed M. Kotb, Mohie A. M. Haridy, Seddik Hammad

**Affiliations:** 1 Department of Zoology, Faculty of Science, South Valley University, Qena 83523, Egypt; 2 Institute of Anatomy and Cell Biology, University Medicine Greifswald, Greifswald, Germany; 3 Department of Anatomy and Histology, Faculty of Veterinary Medicine, Assiut University, Assiut, Egypt; 4 Department of Pathology & Clinical Pathology, Faculty of Veterinary Medicine, South Valley University, Qena 83523, Egypt; 5 Molecular Hepatology - Alcohol Associated Diseases, Department of Medicine II, Medical Faculty Mannheim, University of Heidelberg, Mannheim, Germany; 6 Department of Forensic Medicine and Toxicology, Faculty of Veterinary Medicine, South Valley University, Qena 83523, Egypt

**Keywords:** BPF, liver, kidney, oxidative stress, rats, mercuric chloride

## Abstract

Bioactive peptides such as bradykinin potentiating factor (BPF), have, anti-oxidative, anti-inflammatory, immunomodulatory and ameliorative effects in chronic diseases and play a potential role in cancer prevention. It is known that the liver and kidney accumulate inorganic mercury upon exposure, which often leads to mercury intoxication in these organs. In this study, we investigated the effect of bradykinin potentiating factor (BPF), a scorpion venom peptide, on mercuric chloride-induced hepatic and renal toxicity in rats. We used 20 adult male Albino rats divided into four equal groups: the first group was injected with saline (control); the second group was administered daily with mercuric chloride (HgCl_2_) for 2 weeks; the third group was administered with BPF twice weekly for 2 successive weeks, while the fourth group was exposed to BPF followed by HgCl_2_. We observed that HgCl_2 _treated rats had a significant increase in serum ALT, AST, ALP, creatinine and urea levels compared to control. Furthermore, HgCl_2 _treated rats showed a marked decrease in total proteins, albumin and uric acids compared to control. The previously studied parameters were not significantly changed in BPF pretreated rats compared to control. Moreover, a significant decrease in the activities of glutathione perioxidase (GSH), superoxide dismutase (SOD), and catalase (CAT), in addition to a significant increase in the level of malondialdehyde (MDA) were observed in hepatic and renal tissues of rats after HgCl_2_ treatment. In contrast, the HgCl_2_/BPF treated rats showed a significant elevation in the activity of GSH, SOD, and CAT accompanied with a significant regression in the level of MDA compared to the HgCl_2_ exposed rats. We conclude that treatment with BPF is a promising prophylactic approach for the management of mercuric chloride-induced hepato- and nephro-toxicities.

## Introduction

Mercuric chloride (HgCl_2_) is a highly toxic and corrosive chemical substance. Inorganic mercury has affinity for plasma proteins, and also attaches to red blood cells. Despite of lack the ability to pass through the blood-brain or placental barriers, it reaches to several other body organs. It has been previously shown that HgCl_2_ poisoning can occur through several routes including inhalation, ingestion, and skin absorption (Goyer and Clarkson, 2001[24]). Several studies were focused on implementation of free radicals and oxidants (such as hydrogen peroxide) in the renal injury induced by HgCl_2_ (Von Burg, 1995[61]; Mahboob et al., 2001[31]). It is also already known that HgCl_2_ also demolishes protective antioxidants, and reduces free radical scavenging systems, such as superoxide dismutase (SOD) and glutathione (GSH) peroxidase (Mahboob et al., 2001[31]; Miller et al., 1991[38]). 

Bradykinin potentiating factor (BPF) works through antigen-receptor signalling pathway. Bradykinin antigen binds to bradykinin B2 receptor and induces nitric oxide (NO) production, upregulates antioxidant cupper/zinc (Cu/Zn-SOD) and magnesium superoxide dismutase (MnSOD) expressions, decreases NADPH oxidase activity. It also inhibits reactive oxygen species (ROS) production, and protects against oxidative cardiomyocyte senescence (Laher, 2014[29]). It has been shown that the venom of the Egyptian scorpion, *Buthus occitanus,* contains a peptide fraction that has a bradykinin potentiating activity (El-Saadani, 2004[20]). BPF has been detected not only in scorpions, but also in snakes and jelly fish venoms (Camargo et al., 2005[14]). The effect of BPF on guinea pig kidney was investigated in *vivo* and *in vitro*. Accordingly, a major effect of BPF on guinea pig kidney was the induction of prostaglandin biosynthesis, which disturbs the glomerular filtration function of the kidney (El-Saadani, 2004[20]). Additionally, repair of burn wounds have been accelerated by using BPF originated from scorpion venom (Camargo et al., 2005[14]). Moreover, application of BPF to Guinea pigs exposed to sublethal irradiation dose accelerated regeneration and cellular repopulation of thymus and spleen. Furthermore, the hematological parameters were not significantly changed compared to non-irradiated animals (Salman, 2002[52]). Recently, cadmium-induced liver and kidney damage was markedly ameliorated after application of scorpion venom fraction of BPF. BPF acts as a potent scavenger of free radicals, thus protects tissues against acute cadmium intoxication (Bekheet et al., 2011[11]). It has been described that pretreatment of BPF significantly attenuated the hematological, biochemical and histopathological changes induced by gentamicin (Bekheet et al., 2013[12]). Recently, the oxidative stress induced by CCl_4_-exposure was reduced after application of scorpion venom-originated BPF (Salman, 2015[54]). The effect of BPF on mercuric chloride-induced oxidative damage in the liver and kidney has not been reported. We hypothesized that bradykinin ameliorates mercuric chloride-induced oxidative stress in hepatic and renal toxicities. 

## Materials and Methods

### Mercuric chloride 

Mercuric chloride (Elnasar Company for Chemical and Pharmaceutical Industries) was dissolved in distilled water and intraperitoneally injected (i.p) at a dose of (0.5mg/kg) once daily for fifteen days (Chmielnicka et al., 1983[15]).

### Isolation of bradykinin potentiating factor

The scorpions, *Buthus occitanus *(collected from Qena governorate-Egypt) were milked using electrical shock (6 volts) at the articular membrane of the telson into a clean dry glass container. The collected venom was lyophilized, freeze-dried and then kept at 10° C (in the dark) till used. BPF isolation and purification were preceded according to previous protocol (Ferreira, 1965[21]). Briefly, venom suspension (1 g in 100 ml of distilled water) was heated in boiling water bath for 5 min. Absolute ethanol (750 ml) was added to the suspension, mixed and centrifuged (2000 rpm) for 60 min and the supernatant was evaporated using rotavapor apparatus (R110, Switzerland, App. No. 85860). Ethanol (100 ml, 90 % conc.) was added to the dried powder for 3 successive times. Ethyl ether was added to the pooled alcoholic solution (4:1). The mixture was centrifuged and the resultant precipitate was dissolved in distilled water (250 ml). Lyophilisation and storage of the solution was done till used. The contraction of Guinea pig ileum was stimulated in presence of synthetic bradykinin (B-3259, Sigma Chemicals Co., St. Louis, U.S.A.) approving the activation of bradykinin fraction in venom. The maximum contraction of Guinea pig ileum was recorded for 2-20 min after application of 0.3 µg/ml of the bradykinin fraction in Tyrodo's solution using oscillography (400 MD2 C. Palmer Bioscience, Washington, U.S.A.). Fifty seconds after venom fraction application, synthetic bradykinin (0.02 µg/ml) was added (Nassar et al., 1990[41]). The isolation of BPF from the scorpion venom was performed as previously mentioned and the LD_50_ was determined according to Meier and Theakston (1986[34]).

### Experimental animals

Twenty adult male Albino rats (weighing 200 ± 10 g) were obtained from laboratory animal house (Qena, Egypt). The animals were kept in plastic cages with wire mesh covers under normal environmental conditions of temperature and humidity. Water and suitable commercial diet were supplied *ad libitum* throughout the experiment period. The rats were equally divided into four groups; each group containing 5 rats. Group 1 received saline. Groups 2, 3, and 4 received intraperiotoneal injection of Mercuric chloride, BPF and BPF-Mercuric chloride, respectively (Table 1(Tab. 1)). The experimental protocol was approved by the experimental animal ethics committee, Faculty of Science, South Valley University, Qena, Egypt. All rats were humanely euthanized 24 h after the last application. 

### Serum and tissue sampling 

Before sacrifice, blood samples was collected in tubes without EDTA, left for about 10 min to coagulate, and then centrifuged for 20 min at 3000 rpm. The serum fraction was extracted and preserved at -80° C until used.

Liver and kidney tissues were homogenized in (10 %, w/v) cold sucrose buffer (0.25 M sucrose, 1 mM EDTA and 0.05 M Tris-HCl, pH 7.4) using Thomas Sci Co. glass-type homogenizer (Teflon pestle). A buffer (1.15 % KCl) was added to obtain (1:10 w/v) whole homogenate. To assay malondialdehyde (MDA), superoxide dismutase (SOD) and catalase (CAT) activities, centrifugation was performed at 18,000 × g (4° C) for 15 min followed by 25,000 × g for 50 min to determine glutathione peroxidase (GSH-Px) activities. The supernatants were kept at -80° C till used for assessment of oxidative stress biomarkers in hepatic and renal tissues. 

### Assessment of biochemical parameters in serum

Aspartate aminotransferase (AST) and Alanine aminotransferase (ALT) determination was carried out by a colorimetric method described (Reitman and Frankel, 1957[49]). Serum Alkaline phosphatase (ALP) was measured using the hydrolyzed phenol method (Kind and King, 1954[28]). Urea was assessed using the diacetyl monoxime according to total urinary excretion method (Toro and Ackermann, 1975[59]). Creatinine was anaylsed using the Jaffe alkaline picrate method (Annino and Giese, 1979[5]). 

### Assessment of perioxidase activity 

Hepatic and renal lipid peroxidation (LP) was measured and expressed in terms of MDA content (Placer et al., 1966[47]). Catalase activity was determined by the method of (Aebi, 1984[2]). Superoxide dismutase and glutathione peroxidase activities were estimated according to (Paoletti and Mocali, 1990[46]), and (Maral et al., 1977[32]), respectively.

### Statistics

The data were analyzed by means of one-way analysis of variance (ANOVA) and presented as mean ± S.E. Statistical analysis was done following Student's t-test. A difference was considered significant when *P* < 0.05.

## Results

### Effect on BPF on biochemical parameters 

**Figure 1:**
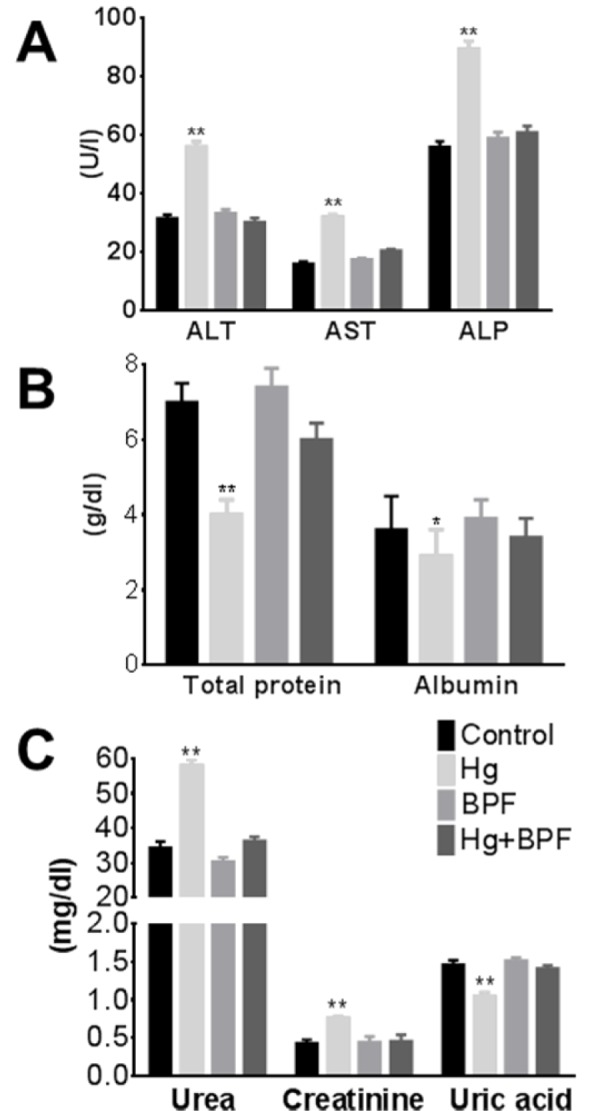
Serum analysis of liver and kidney functions. A) Liver function enzymes e.g. ALT, AST and ALP level indicates that hepatotoxicity is induced by Hg administration. This hepatic injury can be normalized by BPF pre-treatment. B) Both total protein and albumin is significantly decreased in serum of Hg treated rats and reversed by BPF administration. C) Kidney function parameters e.g. urea, uric acid and creatinine suggesting the induction of nephrotoxicity by Hg administration, and the protective effect of BPF. BPF: bradykinin potentiating factor; ALT: alanine aminotransferase; AST: aspartate aminotransferase; ALP: alkaline phosphatase; Hg: Mercuric chloride. Bars are means ± SEM (n = 5). *P < 0.05 and **P < 0.01 vs. control group.

To assess the impact of HgCl_2_ and BPF on liver functions, we measured the serum level of commonly used biomarkers for hepatic toxicity, namely, ALT, AST, ALP and albumin. The serum levels of ALT, AST and ALP in HgCl_2_ treated rats were significantly elevated as compared to the control group. The serum enzyme activities of ALT, AST and ALP in groups 3 and 4 were not significantly changed when compared to the control group (Figure 1A(Fig. 1)). The total protein and albumin level in serum is a hallmark for kidney efficiency. In the present study we found that the total protein and albumin in sera of rats in group 2 were decreased (p < 0.01) as compared to the control group (Figure 1B(Fig. 1)). No significant changes were observed in the serum levels of total protein and albumin in group 3 and 4 as compared to the control group (Figure 1B(Fig. 1)). We found that serum creatinine and urea levels were significantly increased (p < 0.05) in group 2 (was administered daily with HgCl_2_ for 2 weeks) compared to control values, in contrast levels of uric acid were significantly decreased (Figure 1C(Fig. 1)). There are no statistical significant differences in levels of creatinine, urea and uric acid in group 4 when compared to control group (Figure 1C(Fig. 1)). The hepatic and renal biochemical parameters were significantly increased in HgCl_2-_inoculated group; in contrast, these parameters were corrected to control levels when BPF were applied before the exposure to HgCl_2. _These results indicate that a BPF pretreatment normalizes both liver and kidney functions.

### Lipid peroxidation and antioxidant activities

**Figure 2:**
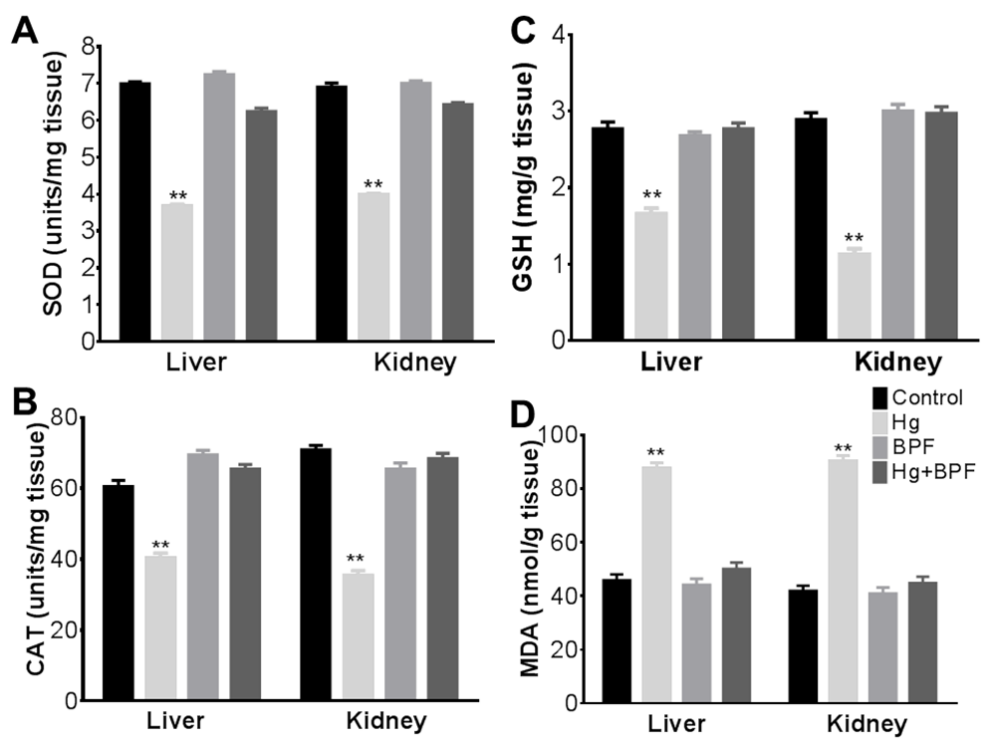
Liver and kidney tissue analysis. Levels of tested anti-oxidant parameters in both liver and kidney are significantly decreased in Hg treated rats compared to control group A) superoxide dismutase (SOD), B) catalase (CAT), C) glutathione (GSH) and D) malondialdehyde (MDA). This reduction is completely abolished by BPF pretreatment. Bars are means ± SEM (n = 5). **P < 0.01 vs. control group

The data of lipid peroxidation, CAT, GSH-Px and SOD activities in the hepatic and renal tissues are summarized in (Figure 2(Fig. 2)). A significant decreased in the level of GSH-Px and SOD activities was detected in the liver (p < 0.05) and kidneys (p < 0.05) of rats in group 2 in comparison to the control group, however GSH-Px and SOD activities were significant higher in group 4 than group 2 (Figure 2A and C(Fig. 2)). MDA was significantly increased (p < 0.05) in the hepatic and renal tissues of rats in group 2. The MDA concentration in HgCl_2_/BPF was significantly decreased in comparison with rats treated with HgCl_2_ (Figure 2D(Fig. 2)). Moreover, CAT activity was significantly decreased in both liver and kidneys of rats in group 2 in comparison to control. The activity of CAT was significantly increased in group 4 when compared to group 2 (Figure 2B(Fig. 2)). These results indicate that a BPF pretreatment ameliorates the HgCl_2_-induced oxidative stress in liver and kidney. 

## Discussion

Mercury is a widely produced in the industry, and can cause serious health hazards. The source of mercury is mainly the environmental pollution by industrial wastes. Early HgCl2-induced nephrotoxicity exacerbates the biochemical imbalance and accelerates hepatotoxicity (Merzoug et al., 2009[35]; Mesquita et al., 2016[36]). In the present study, although HgCl2 was given in small dose (1 mg/kg), liver functions were detrimentally altered. Heavy metals cytotoxicity occurs through membranous damage (Anuradha and Krishnamoorthy, 2012[6]) and a variety of cytoplasmic enzymes of hepatocytes are secreted into the blood stream. Hence, serum enzymes such as ALP, ALT and AST are mainly monitored for the evaluation of hepatic dysfunction and damage. Mercuric intoxication has been recorded to cause significant increases in ALP, ALT and AST activities (Bando et al., 2005[9]; Jagadeesan and Pillai, 2007[26]; Oriquat et al., 2012[44]; Godoy et al., 2013[23]; Abdel-Wareth et al., 2014[1]; Vartak et al., 2016[60]). Changes in lipid and protein metabolisms are important markers of hepatic tissue integrity and function. Albumin is synthesized by the liver and most often transports or binds drugs or chemicals. In the present study, total protein and albumin levels decreased significantly in the HgCl_2_-treated rats. It is suggested that exposure to HgCl_2_ could influence protein synthesis and/or metabolism in the liver. Moreover, the decreased albumin level could be a consequence of the impact of mercuric on albumin molecule, since albumin possesses a free sulfhydryl group on a terminal cysteinyl residue to which mercuric ions can bind (Mohamed et al., 2010[39]). 

The kidney maintains the blood creatinine in a normal range. Creatinine has been found to be a fairly reliable indicator of kidney function. Elevated creatinine level signifies impaired kidney function or disease. Abnormally high levels of creatinine warn of possible malfunction or failure of the kidneys. It is for this reason that standard kidney function test is the routinely blood test to check the amount of creatinine in the blood. The higher levels of urea and creatinine are clearly reflected progressing renal insufficiency in Albino rats injected with mercuric chloride (Oriquat et al, 2012[44]). Mercury probably impaired hepatic and renal functions through both vasoconstriction and a direct cytotoxic effect on podocyte cells (foot processes effacement and cells detachment) (Barregard et al., 2010[10]; Girardi and Elias, 1993[22]). Besides, the detrimental effect might be attributed to its accumulation in the renal tissues. 

HgCl_2_ produced a typical pattern of hepatotoxity and nephrotoxicity characterized by marked increase in serum creatinine, blood urea and serum ALT, AST and ALP activities. The elevation in the serum activity of ALT, a liver cytoplasmic enzyme, indicates necrotic and hepatic lesions. Also, HgCl2 showed not only a significant elevation in AST activities, but a significant decline in the ALP activity (El-Demerdash, 2001[19]; Reus et al., 2003[51]; Sharma et al., 2002[57]). ALP activity decreased and increased in acute and chronic exposures respectively, of teleost fish to mercuric chloride (Sastry and Sharma, 1980[56]). 

The severity of hepatic and renal failure are related to the degree of intracellular and extracellular oxidative stress, in which it depends on the excess production of free radical coupled with low concentration of antioxidants (Godoy et al., 2013[23]; Oloyede et al., 2013[43]; Massy and Nguyen-Khoa, 2002[33]). Free radical-induced lipid peroxidative damage has played a significant role in the pathogenesis of various liver and kidney diseases. Lipid peroxidation (LP) is assayed indirectly by production of secondary products like a low molecular weight reactive aldehyde malondialdehyde (MDA) and assessment of antioxidant status can be measured by estimating serum superoxide dismutase (SOD).

Mercury toxicity is increased the production of free radicals and hence oxidative stress (Bando et al., 2005[9]; Durak et al., 2010[18]). MDA is the end product of LP and an increase in its level is indicative of peroxidation (Su et al., 2008[58]). In the present study, MDA level was significantly increased in tissues of liver and kidneys of HgCl_2_-treated rats. The injection of HgCl_2_ increased MDA level in various tissues including kidneys and brain (Agarwal et al., 2010[3]; Aslanturk et al., 2014[8]), testis (Kalender et al., 2013[27]) and thyroid gland in a dose-dependent manner (Rao and Chhunchha, 2010[48]). Increment of MDA level induced by HgCl*_2_* is considered indicative for hepatic and renal damage. SOD, CAT, and GSH-Px are essential for the cellular protection against reactive oxygen species (ROS) and other oxidative stress (Morakinyo et al., 2012[40]). SOD is included in the detoxification process to catalyze the dismutation of superoxide radicals to H_2_O_2_ and molecular oxygen (Boujbiha et al., 2009[13]). CAT activity is included in the reduction of H_2_O_2_ to H_2_O and oxygen and in turn cellular protection against oxidative damage produced by H_2_O_2_ and hydroxyl radical (Renugadevi and Prabu, 2010[50]). In the present work, significant decreases of SOD, CAT, and GSH-Px activities in the hepatic and renal tissues of HgCl_2_-treated rats were recorded. Mercuric chloride increases the generation of many endogenous oxidants such as H_2_O_2_ that causes lipid peroxidation. The decreases in antioxidant activities in mercuric intoxication are due to the excess generation of ROS and resultant enhancement in lipid peroxidation (Agarwal et al., 2010[3]). The reduction in the activities of SOD, CAT, and GSH-Px enzymes in the present study might be due to their consumption during the breakdown of free radicals and the accumulation of superoxide radicals and H_2_O_2_ or due to the inhibition of these enzymes by free radicals (Aslanturk et al., 2014[8]; Othman et al., 2014[45]; Rao and Chhunchha, 2010[48]). Hepatic functions were also impaired by administration of mercury suggesting its induction of oxidative stress in the treated rats. This oxidative stress included augmentation of lipid peroxidation as well as inhibition of the antioxidant enzyme activities such as GSH, SOD and CAT enzymes. Lipid peroxidation was increased as expressed in elevation of MDA and depression in the activities of GSH, SOD and CAT enzymes in kidney and liver in mercuric chloride-treated rats. 

The biochemical assays in the present study demonstrated that exogenous BPF from scorpion venom reduced the HgCl_2_-induced oxidative stress. Hence, the treatment with BPF restored the activity of antioxidant in streptozotocin- induced hyperglycemic rat (Mikrut et al., 2001[37]). Also, kinin infusion protected against salt-induced renal dysfunction (Oeseburg et al., 2009[42]). Moreover, it has also been shown that after malathion exposure, the total globulin concentration, IgG, IgM, total immunoglobulins and circulatory immune complexes were significantly decreased. In contrast, after injection of BPF, bone marrow and splenic changes and peripheral blood elements were recovered to control levels and the elevated proinflammatory markers (IL-2, IL-4 and TNF-α), total plasma peroxide and oxidative stress index were reduced associated with an increase in total antioxidant capacity (Ahmed, 2012[4]). The present findings indicated the important role of kinins in control the development of oxidative stress. However, the mechanism by which kinins could chelate free radical and inhibit peroxide production, as well as SOD, CAT and GSH activities *in vivo*, is not clearly defined. The effects of bradykinin are mediated via local stimulation of prostaglandin synthesis (Dietze et al., 1978[17]; Camargo et al., 2012[14]). The decreased level of MDA, observed after bradykinin administration, may thus be due to a reduction in free radical production. 

Additionally, CCl_4_-induced liver injury was protected by bradykinin infusion, as revealed by a dramatic reduction of serum liver enzyme levels in bradykinin- treated rats and in turn decreased hepatic injury (Sancho-Bru et al., 2007[55]). BPF activates the hepatic glycogen synthetase that enhances hepatic glycogen synthesis that potentiates integrity of hepatocytes and detoxification capability. Moreover, it increases immunoglobulin production either in bone marrow or plasma cells and either directly or through the cytokine regulation (Liu et al., 1992[30]). After irradiation of rats, significant increases in renal MDA and advanced oxidation protein product and serum urea and creatinine levels associated with significant decrease in renal GSH and uric acid levels. After injection of BPF all these parameters were corrected to control levels (Ashry et al., 2012[7]). Bradykinin inhibits renal fibrosis by increasing the NO production, suppression of TGFβ1 expression and mitogen-activated protein kinase (ERK and p38) phosphorylation (Hagiwara et al., 2004[25]).

Thus, it is clear that administration of BPF is effective in reducing biochemical alterations and oxidative stress caused by HgCl_2_ in rat liver and kidneys. BPF had free radical-scavenger effect and/or an enhancing effect on the antioxidant capacity of the body. Therefore, activation of the kallikrein-kinin system is a promising prophylactic approach for the management of subacute hepatic and renal toxicities.

## Figures and Tables

**Table 1:**
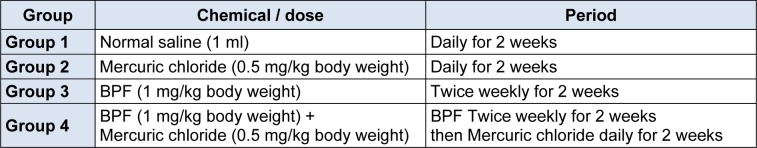
Experimental design
